# Supplementary material for: Predicted Membrane-Associated Domains in Proteins Encoded by Novel Monopartite Plant RNA Viruses Related to Members of the Family Benyviridae
Source: Int J Mol Sci. 2023 Jul 29;24(15):12161. doi: 10.3390/ijms241512161 (PMC10418960; doi:10.3390/ijms241512161)
Supplement: Supplementary file 1 [file ijms-24-12161-s001.zip › 2023-Supplementary Figure S1.pdf]

***Dactylorhiza hatagirea* beny-like virus**

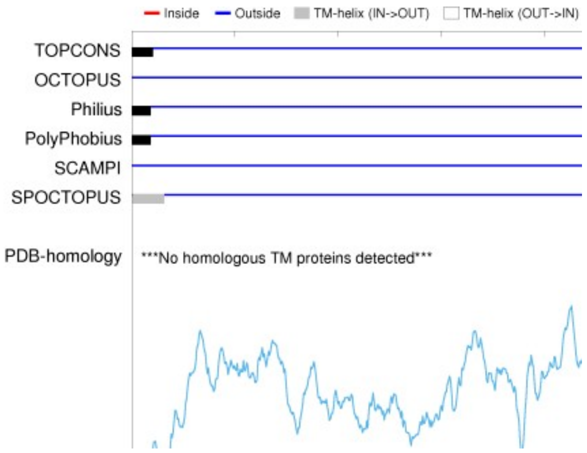

***Gymnadenia rhellicani* TR10147**

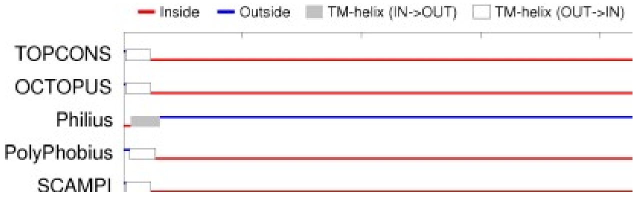

***Ophrys sphegodes* VLRA**

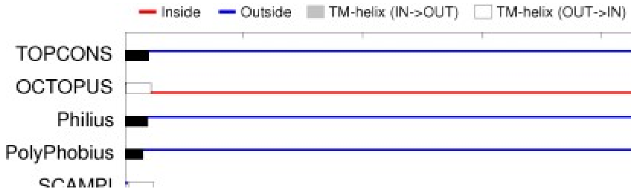

***Ophrys fusca* VLRA**

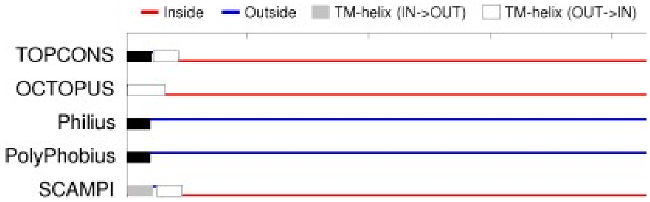

***Sarcodes sanguinea* VLRA**

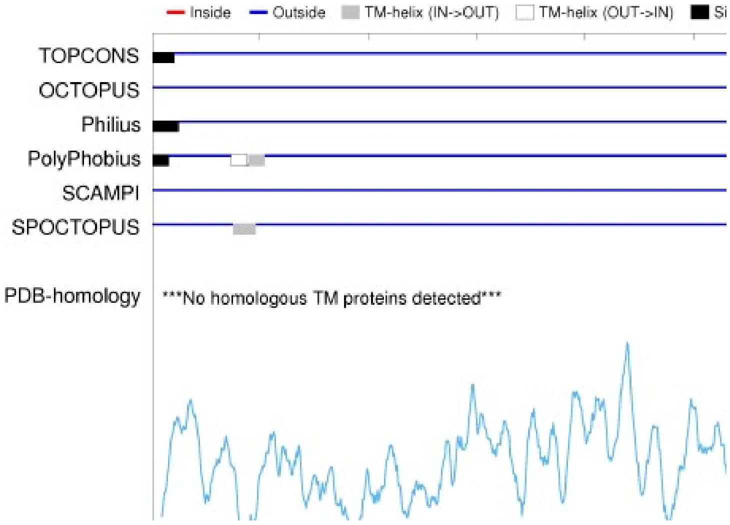

***Striga hermonthica* VLRA**

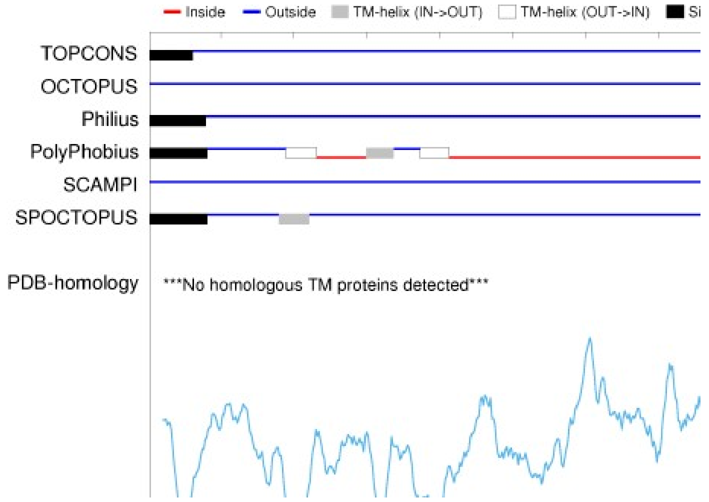

***Silene dioica* VLRA**

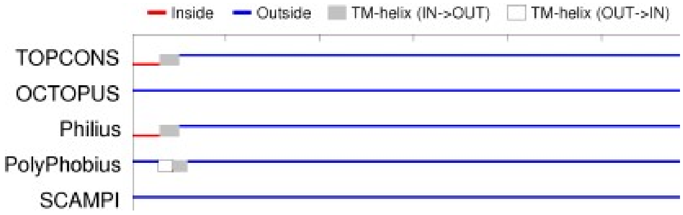

***Rhyncholacis* cf. penicillata Rhyc16**

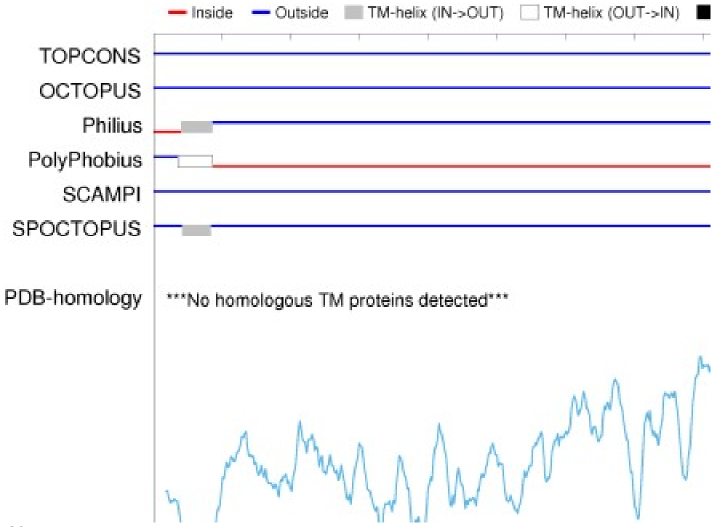

***Rhyncholacis* cf. penicillata Rhyc2783**

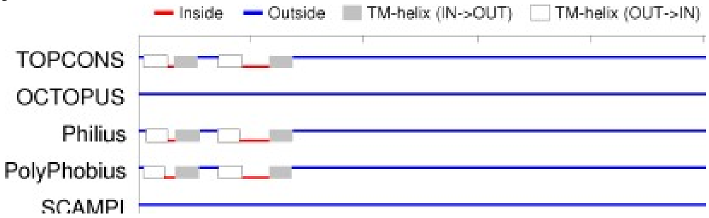

Supplementary Figure S1. Prediction of replicase membrane-spanning segments in the N-terminal regions of the reclovirid replicases using TOPCONS software. See Materials and Methods for details.
