# Supplementary material for: Predicted Membrane-Associated Domains in Proteins Encoded by Novel Monopartite Plant RNA Viruses Related to Members of the Family Benyviridae
Source: Int J Mol Sci. 2023 Jul 29;24(15):12161. doi: 10.3390/ijms241512161 (PMC10418960; doi:10.3390/ijms241512161)
Supplement: Supplementary file 1 [file ijms-24-12161-s001.zip › 2023-Supplementary Figure S2.pdf]

***Sarcodes_sanguinea* VLRA (ORF2, 196aa)**

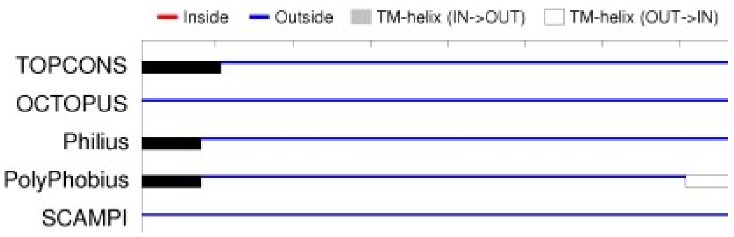

***Atriplex_prostrata* VLRA (ORF2, 140aa)**

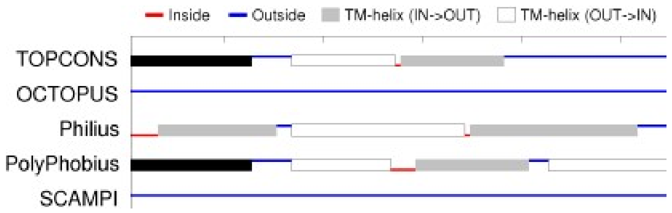

***Atriplex_prostrata* VLRA (ORF3, 167aa)**

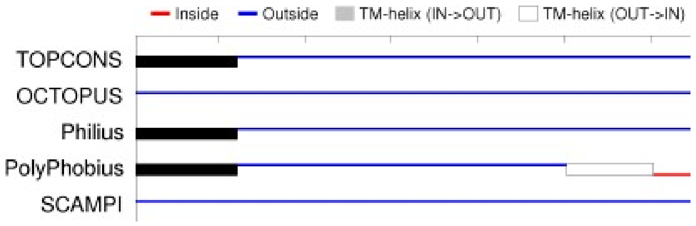

2063723-***Leontopodium_alpinum* VLRA (ORF2, 150aa)**

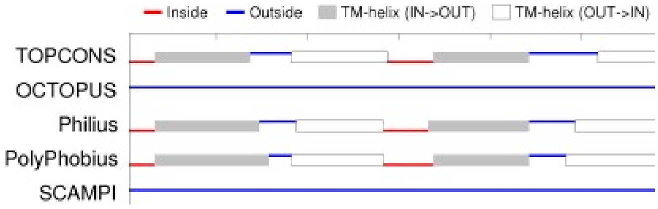

2063723-***Leontopodium_alpinum* VLRA (ORF3, 249aa)**

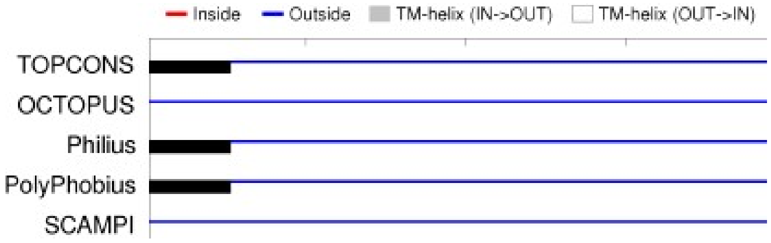

2063722-***Leontopodium_alpinum* VLRA (ORF2, 332aa)**

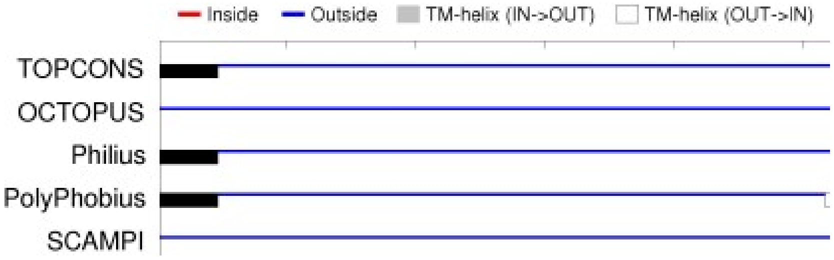

Scutellaria_montana **VLRA (ORF2, 58aa)**

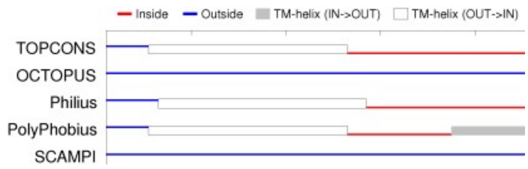

Scutellaria_montana **VLRA (ORF4, 46aa)**

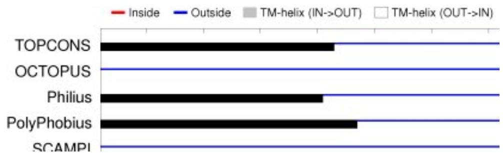

Silene dioica **VLRA (ORF2, 525aa)**

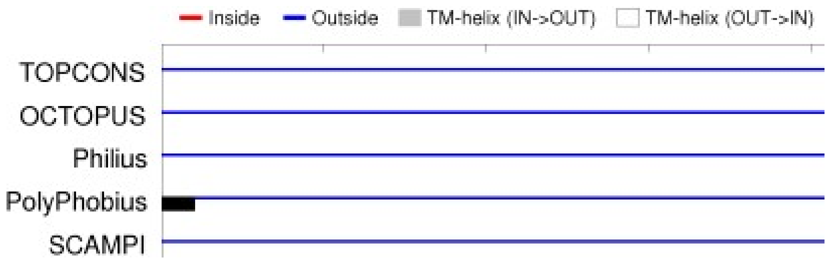

Rhyncholacis **cf. penicillata Rhyc16 VLRA (ORF2, 357aa)**

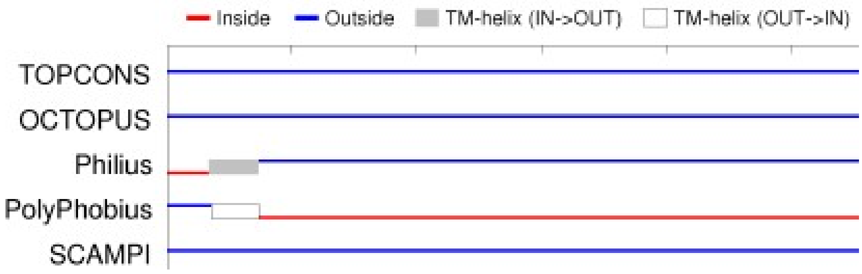

Cistanche tubulosa **VLRA (ORF2, 225aa)**

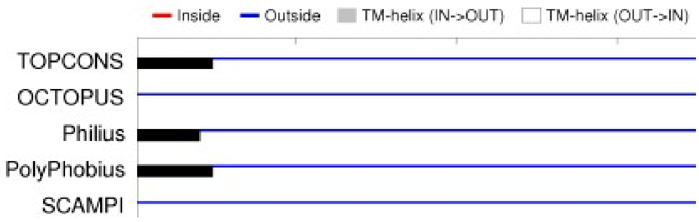

Melampyrum roseum **VLRA (ORF2, >227aa)**

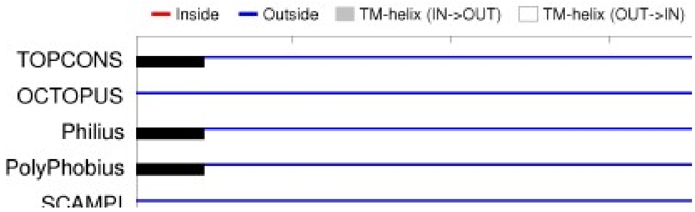

***Striga hermonthica* VLRA (ORF2, 280aa)**

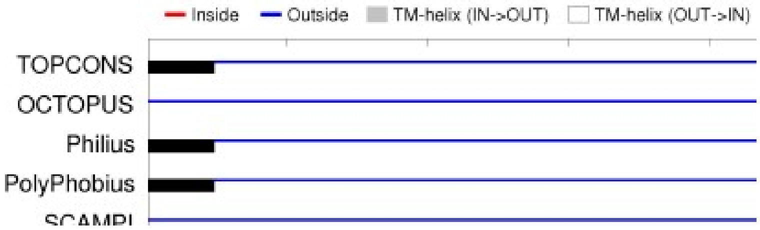

***Coriandrum sativum* VLRA (ORF2, 323aa)**

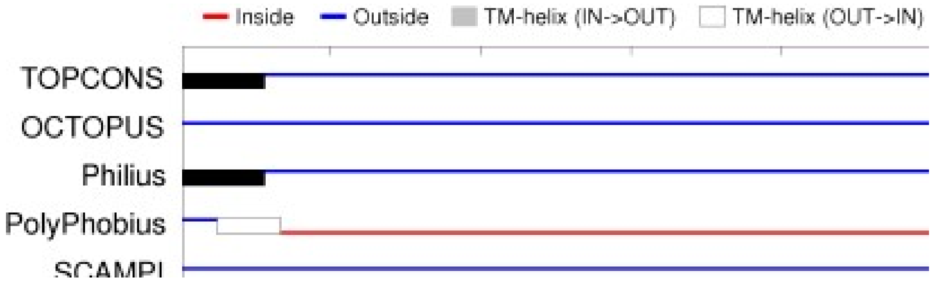

***Camellia reticulata* VLRA (ORF2, 132aa)**

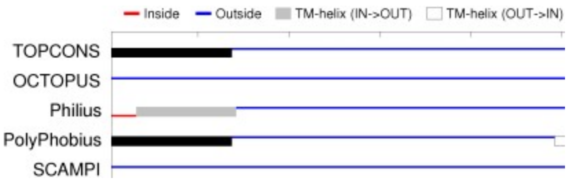

***Viscum album* VaGs28290 VLRA (ORF2, 241aa)**

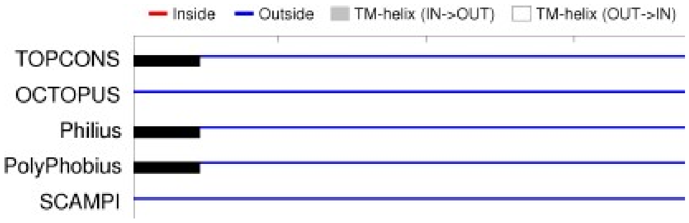

***Viscum album* VaGs14604 VLRA (ORF2, 234aa)**

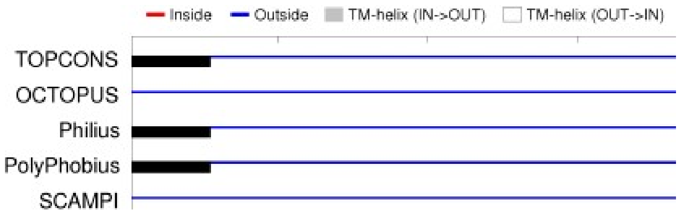

***Viscum album* VaGs14604 VLRA (ORF3, 54aa)**

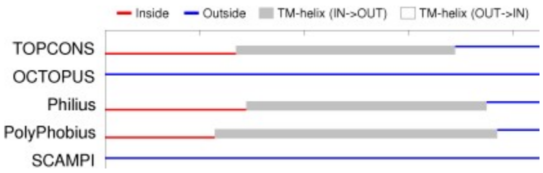

Arceuthobium sichuanense virus 3 **(ORF2, 265aa)**

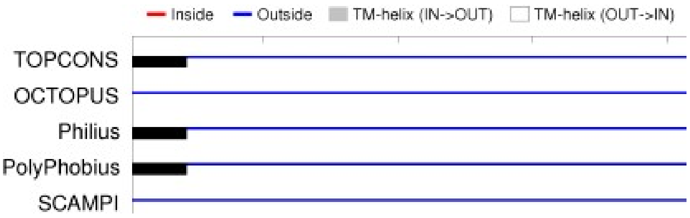

Arceuthobium sichuanense virus 3 **(ORF3, 35aa)**

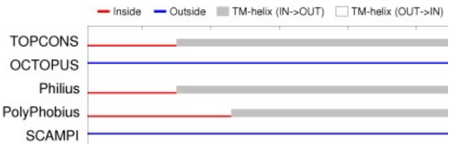

Dactylorhiza hatagirea **beny-like virus (ORF2, 137aa)**

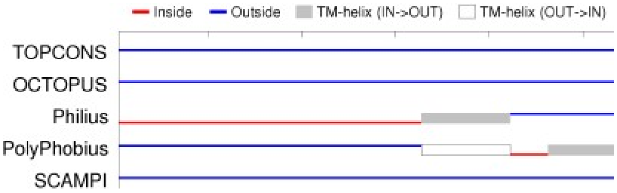

Vicia faba **VLRA (ORF2, 477aa)**

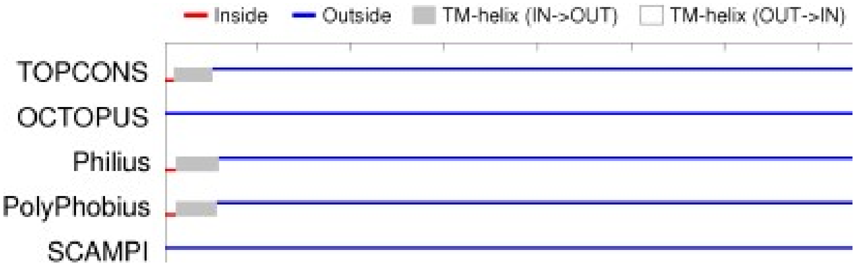

Astragalus canadensis **VLRA (ORF2, 668aa)**

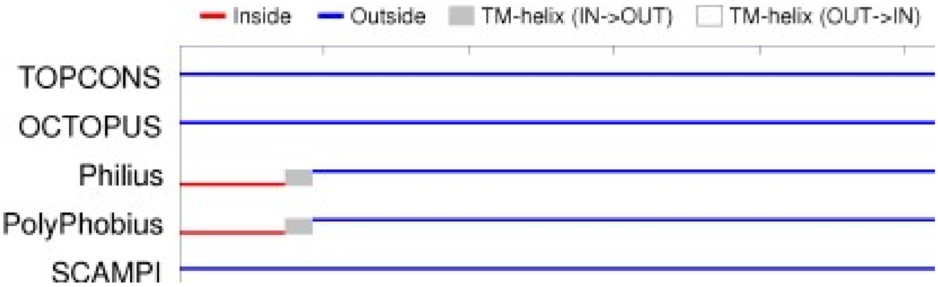

Daiswa yunnanensis **VLRA (ORF2, 110aa)**

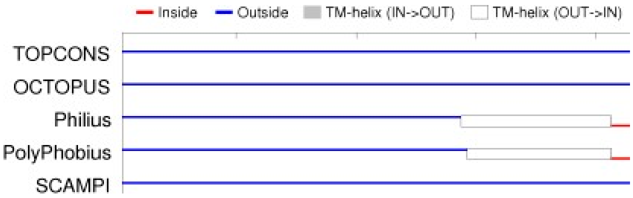

***Daiswa yunnanensis* VLRA (ORF3, 41aa)**

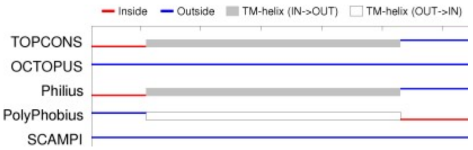

***Gymnadenia rhellicani* TR101479 (ORF2, 145aa)**

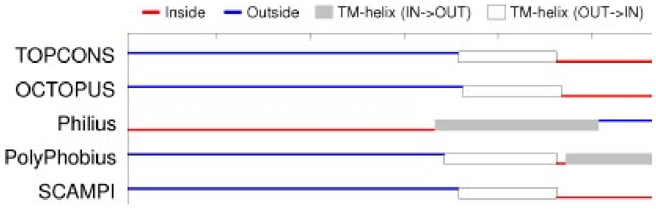

***Gymnadenia rhellicani* TR40174 (ORF2, 63aa)**

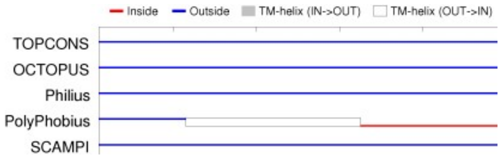

***Gymnadenia rhellicani* TR40174 (ORF3, 137aa)**

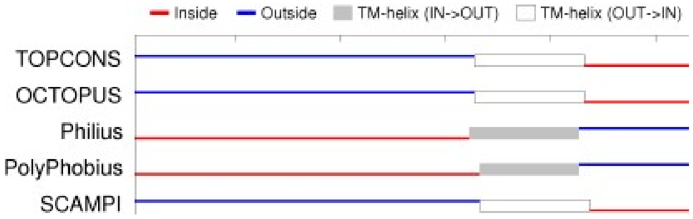

***Carrot associated RNA virus 1* (ORF2, >167aa)**

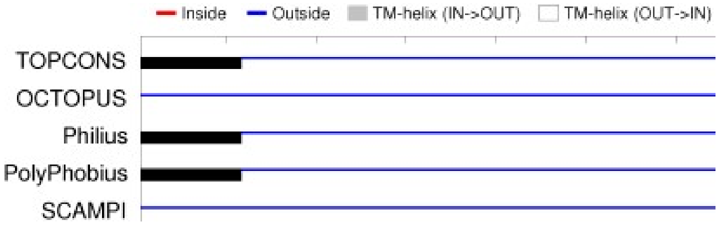

***Red clover RNA virus 1* (ORF2, 112aa)**

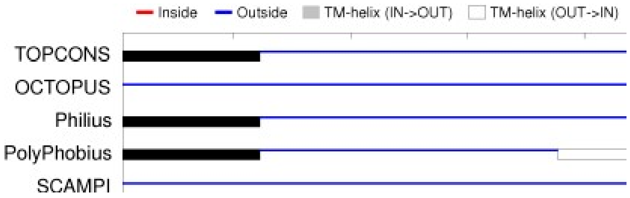

***Red clover RNA virus 1* (ORF3, 66aa)**

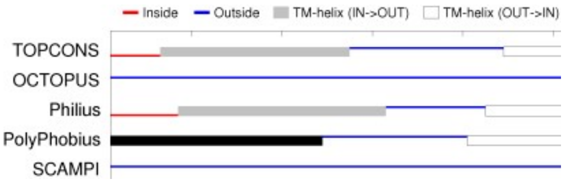

Platanthera guangdongensis **VLRA (ORF2, 137aa)**

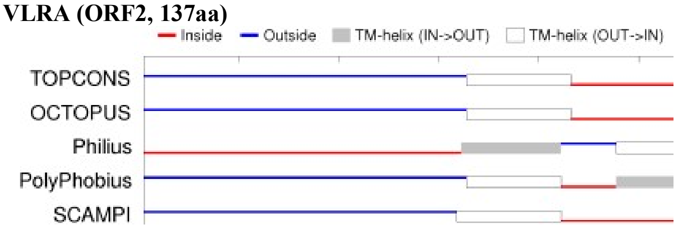

Ophrys sphegodes **VLRA (ORF2, 138aa)**

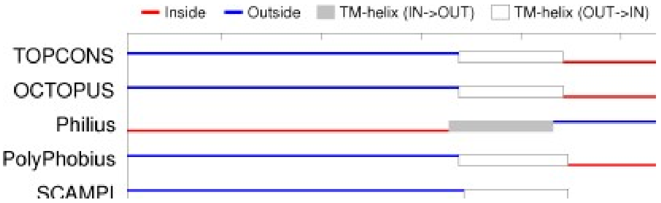

Ophrys fusca **VLRA (ORF2, 137aa)**

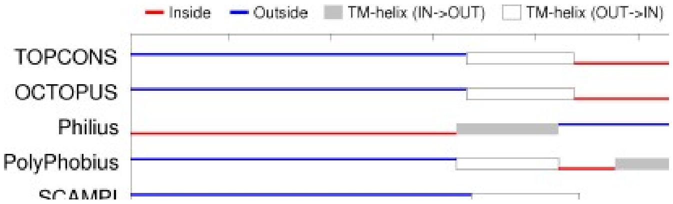

Rhyncholacis **cf. penicillata Rhyc2783 (ORF2, 313aa)**

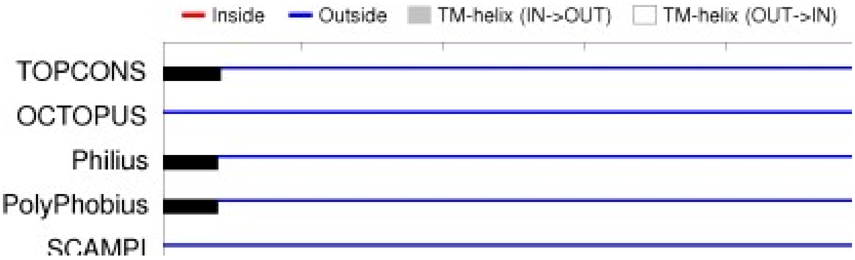

Supplementary Figure S2. Prediction of membrane-spanning segments in the non-replicative proteins of the reclovirids using TOPCONS software. See Materials and Methods and Supplementary Table 1 for details.
