# Supplementary material for: Predicted Membrane-Associated Domains in Proteins Encoded by Novel Monopartite Plant RNA Viruses Related to Members of the Family Benyviridae
Source: Int J Mol Sci. 2023 Jul 29;24(15):12161. doi: 10.3390/ijms241512161 (PMC10418960; doi:10.3390/ijms241512161)
Supplement: Supplementary file 1 [file ijms-24-12161-s001.zip › 2023-Supplementary Table S1.pdf]

Table S1. Genome organization of reclovirids

Hosts of viruses and VLRAs belonging to reclovirids (order and family)	RNA assembly size (nts)	Accession number (NCBI)	Positions and properties of genes and proteins		
			ORF1 replicase (membrane segments)	ORF2 (size, presence of Zn-f and membrane- embedded blocks)	ORF3 (size, presence of peculiar amino acid blocks)
Dactylorhiza hatagirea beny-like virus; *Asparagales; Orchidaceae*	7749	BK013327	207..7058 (+HYDR)	7167..7580 (+HYDR) (137aa) Zn-f: CX(3)CX(10)CX(3)C	ND*
Gymnadenia rhellicani TR10147 VLRA; *Asparagales; Orchidaceae*	7915	GHXH01324014	230..7111 (+HYDR)	7230..7667 (+HYDR) (145aa) Zn-f: CX(3)CX(10)CX(3)C	7681..7806 (41aa)
Gymnadenia rhellicani TR40174 VLRA; *Asparagales; Orchidaceae*	1320	GHXH01128483	<1..512	565..756 (+HYDR) (63aa)	630..1043 (+HYDR) (137aa) Zn-f: CX(3)CX(10)CX(3)C
Platanthera guangdongensis VLRA; *Asparagales; Orchidaceae*	1756	SRX14997078	<1..1005	1090..1504 (+HYDR) (137aa) Zn-f: CX(3)CX(10)CX(3)C	ND*
Ophrys sphegodes VLRA; *Asparagales; Orchidaceae*	8005	GHXJ01414654	235..7281 (+HYDR)	7359..7775 (+HYDR) (137aa) Zn-f: CX(3)CX(10)CX(3)C	ND*
Ophrys fusca VLRA; *Asparagales; Orchidaceae*	7998	GHXI01129489	231..7274 (+HYDR)	7294..7705 (+HYDR) (137aa) Zn-f: CX(3)CX(10)CX(3)C	ND*
Daiswa yunnanensis VLRA; *Liliales*; *Melanthiaceae*	2807	GFOY01013898	<1..2107	2145..2477 (+HYDR) (110aa) Zn-f: CX(3)CX(9)CX(3)C	2485..2610 (+HYDR) (41aa)
Sarcodes sanguinea VLRA; *Ericales; Ericaceae*	7546	SERM-2010905**	274..6597 (+HYDR)	6730..7320 (+HYDR) (196aa) Zn-f: HXHX(4)CX(3)H	ND*
Camellia reticulate VLRA; *Ericales; Theaceae*	6434	GEER01003429	83..5974	6039..6434 (+HYDR) (132aa) Zn-f: CX(3)CX(8)CX(3)C	ND*
Leontopodium alpinum VLRA; *Asterales; Asteraceae*	7735	DOVJ-2063723**	121..6738	6812..7264 (+HYDR) (150aa)	6820..7569 (+HYDR) (249aa) Zn-f: CX(3)CX(10)CX(3)C
Leontopodium alpinum VLRA; *Asterales; Asteraceae*	7388	DOVJ-2063722**	<1..6198	6253..7251 (+HYDR) (332aa) Zn-f: CX(3)CX(7)CX(3)C	ND*
Scutellaria montana VLRA; *Lamiales; Lamiaceae*	2402	ATYL-2017654**	<1..1025	1099..1275 (+HYDR) (58aa)	**ORF3**: 1307..1402 (31 aa); **ORF4**: 1414..1554 (+HYDR) (46 aa); **ORF5**: 1887..2216 (109 aa)
Cistanche tubulosa VLRA; *Lamiales; Orobanchaceae*	1968	GJRS01079843	<1..994	1087..1764 (+HYDR) (225aa) Zn-f: CX(10)CX(10)CX(7)C	ND*
Melampyrum roseum VLRA; *Lamiales; Orobanchaceae*	2174	IADV01103213	<1..1430	1493..>2174 (+HYDR) (>227aa) Zn-f: CX(2)HX(10)CX(9)H	ND*
Striga hermonthica VLRA; *Lamiales; Orobanchaceae*	8023	ICPL01009187	58..6909 (+HYDR)	7021..7863 (+HYDR) (280aa)	ND*
Carrot associated RNA virus 1; *Apiales; Apiaceae*	1304	OM419188 SRX13122999	<1..688	801..>1304 (+HYDR) (>167aa)	ND*
Coriandrum sativum VLRA; *Apiales; Apiaceae*	8089	GGPN01001998	228..6824	6951..7922 (+HYDR) (323aa) Zn-f: HX(5)CX(29)CX(4)C	ND*

Atriplex prostrata VLRA; *Caryophyllales; Chenopodiaceae*	8004	AAXJ-2011446** EPVF-2046303**	337..7316	7346..7778 (+HYDR) (140aa)	7388..7892 (+HYDR) (167aa) Zn-f: CX(3)HX(4)CX(2)C
Silene dioica VLRA; *Caryophyllales; Chenopodiaceae*	9583	GFCG01071918	336..7416 (+HYDR)	7857..9435 (+HYDR) (525aa) Zn-f: CX(3)CX(7)CX(3)C	ND*
Arceuthobium sichuanense virus 3; *Santalales; Viscaceae*	7340	BK059270	61..6276	6341..7138 (+HYDR) (265aa) Zn-f: CX(3)CX(11)CX(3)C	7157..7264 (+HYDR) (35aa)
Viscum album VLRA; *Santalales; Viscaceae*	2781	GJLG01028288	<1..1812	1884..2609 (+HYDR) (241aa) Zn-f: CX(3)CX(11)CX(3)C	ND*
Viscum album VLRA; *Santalales; Viscaceae*	1076	GJLG01014603 SRX12291946	<1..109	137..841 (+HYDR) (234aa) Zn-f: CX(3)CX(11)CX(3)C	903..1067 (+HYDR) (54aa)
Rhyncholacis cf. penicillata Rhyc16; *Malpighiales; Podostemaceae*	7872	ICSC01000014	206..6595 (+HYDR)	6596..7669 (+HYDR) (357aa) Zn-f: CX(3)CX(7)CX(3)C	ND*
Rhyncholacis cf. penicillata Rhyc2783; *Malpighiales; Podostemaceae*	8275	ICSC01056734	153..7082 (+HYDR)	7160..8101 (+HYDR) (313aa) Zn-f: CX(8)CX(5)HX(5)C	ND*
Vicia faba VLRA; *Fabales; Fabaceae*	2625	GISP01006645 SRX10153333	<1..924	986..2417 (+HYDR) (477aa) Zn-f: CX(3)CX(7)CX(3)C	2544..2615 (+HYDR) (26aa)
Astragalus canadensis VLRA; *Fabales; Fabaceae*	2770	GGNK01006218	<1..704	744..2748 (+HYDR) (668aa) Zn-f: CX(3)CX(7)CX(3)C	ND*
Red clover RNA virus 1; *Fabales; Fabaceae*	7190	MG596242	103..6639	6692..7030 (+HYDR) (112aa) Zn-f: CX(3)CX(8)CX(3)C	6771..6971 (+HYDR) (66aa)
sequence ND_128787***	3764	-	<1..3201	3317..>3764 (+HYDR)	ND*

*ND – not determined; ** - 1KP database; *** - riboviria.org database; (+HYDR) means the presence of membrane spanning segment(s)
